# Similarity-Based Remaining Useful Lifetime Prediction Method Considering Epistemic Uncertainty

**DOI:** 10.3390/s23239535

**Published:** 2023-11-30

**Authors:** Wenbo Wu, Tianji Zou, Lu Zhang, Ke Wang, Xuzhi Li

**Affiliations:** 1 University of Chinese Academy of Sciences, Beijing 101408, China; wuwenbo@csu.ac.cn (W.W.); zoutianji@csu.ac.cn (T.Z.); wangke@csu.ac.cn (K.W.); xzhli@csu.ac.cn (X.L.); 2 Technology and Engineering Center for Space Utilization, Chinese Academy of Sciences, Beijing 100094, China; 3 The Key Laboratory of Space Utilization, Beijing 10094, China

**Keywords:** remaining useful lifetime, epistemic uncertainty, uncertainty theory, similarity measure, degradation trajectories

## Abstract

Measuring the similarity between two trajectories is fundamental and essential for the similarity-based remaining useful life (RUL) prediction. Most previous methods do not adequately account for the epistemic uncertainty caused by asynchronous sampling, while others have strong assumption constraints, such as limiting the positional deviation of sampling points to a fixed threshold, which biases the results considerably. To address the issue, an uncertain ellipse model based on the uncertain theory is proposed to model the location of sampling points as an observation drawn from an uncertain distribution. Based on this, we propose a novel and effective similarity measure metric for any two degradation trajectories. Then, the Stacked Denoising Autoencoder (SDA) model is proposed for RUL prediction, in which the models can be first trained on the most similar degradation data and then fine-tuned by the target dataset. Experimental results show that the predictive performance of the new method is superior to prior methods based on edit distance on real sequence (EDR), longest common subsequence (LCSS), or dynamic time warping (DTW) and is more robust at different sampling rates.

## 1. Introduction

Man-operated spacecraft, ranging from the space shuttle to the deep space exploration vehicle, increasingly operate for extended durations. The space station has been in orbit for decades. As an indispensable energy storage device, lithium batteries are one of the most critical components of spacecraft. In order to guarantee the reliability and safety of spacecraft, it is imperative to implement prognostic and health management (PHM) technologies for lithium batteries. RUL prediction plays a pivotal role in PHM by arranging necessary maintenance in advance to prevent future catastrophic breakdowns [1]. Techniques for RUL prediction can be broadly categorized into two categories: data-driven and physical model-based [2]. The physical model-based method requires sufficient knowledge of a system’s failure mechanisms to build a mathematical description of the system’s degradation process [3,4]. Nevertheless, obtaining a comprehensive understanding of the physical mechanisms for component degradation is a significant challenge. On the other hand, the data-driven method does not require a precise comprehension of the nature of degradation. In the discipline of RUL, implementing deep learning techniques, such as CNN [5] and RNN [6], has been tremendously successful. Conversely, deep learning methodologies require a substantial amount of training data. A lack of adequate samples exacerbates the difficulty associated with training models and presents the risk of overfitting, which could deteriorate prediction accuracy. In engineering practice, especially for aerospace products, adequate failure data are often difficult to obtain due to expensive products, high test costs, and extended test cycles. In conclusion, while deep learning performs well on large sample datasets, it is not well suited for small samples. Transfer learning and reinforcement learning have been shown to have strong performance and effectiveness in dealing with the problem of insufficient fault data. Presently, various transfer learning methods, including auto-encoder [7], consensus self-organizing models [8], and convolutional transfer networks [9], have been successfully applied to RUL prediction. Wang et al. [10] proposed a transfer learning and ensemble learning method that can improve RUL prediction accuracy with small data. Sun et al. [11] proposed a domain-adaptive adversarial network for RUL prediction of bearings under different operating conditions. The main idea of transfer learning is to utilize similar information from the source dataset to improve task performance in the target dataset. Given that the degradation trajectory of batteries can be categorized as a form of time series data, similarity measures such as dynamic time warping (DTW), and longest common subsequences (LCSS) can be used to extract similar information about degradation trends for transfer learning. Mao et al. [12] proposed a RUL prediction method based on transfer learning and the DTW method; the DTW algorithm can extract more similar degradation sequences by considering global trend information, making the prediction results more accurate. Zhu et al. [13] utilized a multi-layer perception network and maximum mean discrepancy (MMD) distance to realize the RUL prediction across different working conditions. Furthermore, typical similarity metrics such as edit distance with real penalty (ERP), edit distance on real sequence (EDR), and matching matrix can also be utilized to quantify the similarity between trajectories [14,15,16,17,18,19,20]. After selecting the degraded trajectories with the highest similarity, their data can be used to pre-train the transfer model and further adjust the parameters according to the measured data, thus realizing the lifetime prediction.

**Figure 1:**
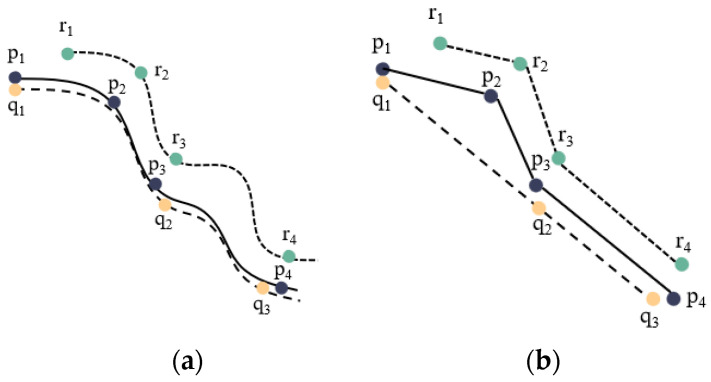
The trajectories P, Q, and R are depicted using two different representations: real movement (**a**) and linear interpolation (**b**).

In these studies, similarity was defined as the distance between any two degradation trajectories. They make the simple assumption that the location of the trajectory is determined. However, the location of degradation trajectories may be uncertain in a general and realistic scenario because they are randomly sampled from the actual continuous paths at random intervals. There will be significant epistemic uncertainty regarding the trajectory’s shape at low and heterogeneous data sampling rates [14]. In Figure 1, we provide an example where the sampled trajectory is discretized into a combination of multiple sampling points at various sampling frequencies, while the original degradation trajectory is represented as a continuous curve. The difference between the sampled and original trajectories increases with decreasing sampling frequency. At various sampling rates, the shape of the degraded trajectories changed significantly, and the linear interpolation method also resulted in the loss of degradation information between different data points. These uncertainties will ultimately impact the precision of RUL by influencing the measure of similarity between trajectories. Figure 1a depicts a visual representation of three trajectories: P, Q, and R. The little circles inside the figure represent the sampled locations. The depiction of P, Q, and R using linear interpolation is shown in Figure 1b, where the transition between the sampled points is considered a straight line. Obviously, similarity (P,R) > similarity (P,Q) in sampled trajectories, whereas in real trajectories, similarity (P,R) < similarity (P,Q). In a word, uncertainty is inevitable in trajectory similarity measurement, and it is important to accurately account for these sources of uncertainty while estimating the RUL.

Considering the epistemic uncertainty that arises from asynchronous sampling, an uncertainty ellipse model, derived from uncertain theory, is proposed for describing the location uncertainty associated with each sampled point along the degraded trajectory. We characterize the shape parameters of the ellipse with a normal uncertainty distribution built on empirical data. On this basis, we propose a novel and effective similarity measure metric for any two degradation trajectories and take this metric as the similarity between trajectories. Then, batteries that are most similar to the target battery can be selected from the historical database based on the distance metric and transferred to train the SDA-based [21] lifetime prediction models.

In this paper, our main contributions are outlined as follows: (1) The proposed uncertain ellipse model and its corresponding distance calculation method exhibit superior accuracy in determining the similarity between uncertain degradation trajectories resulting from asynchronous sampling. (2) By integrating similarity-based RUL prediction methods with uncertain theory, we propose a novel method for predicting RULs that significantly enhances the accuracy of the predictions.

The rest of this paper is organized as follows: Section 2 presents the proposed method combining uncertain ellipses and the SDA network for RUL prediction. In Section 3, the advantages of the proposed method are illustrated by the synthetic and real battery datasets separately. Section 4 concludes the article and presents future trends.

## 2. Methodology

**Figure 2:**
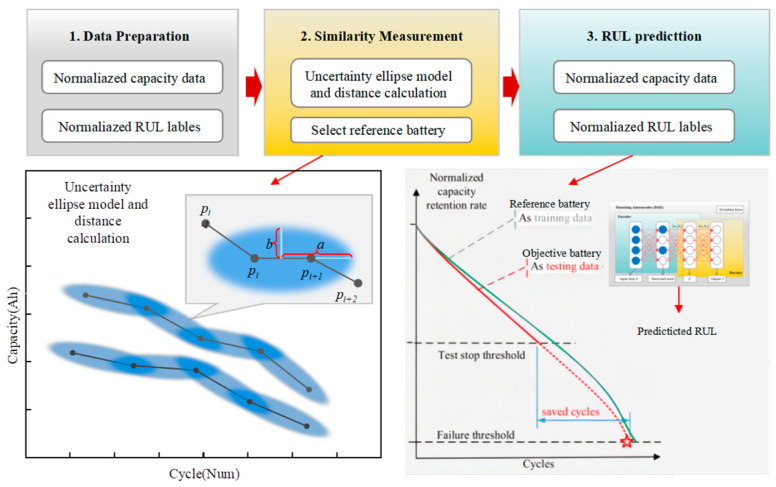
Flow chart of the proposed similarity-based RUL method.

The whole process of the proposed method is depicted in Figure 2. It consists of three main steps: (1) Data preparation, (2) Uncertainty ellipse-based similarity measurement, and (3) SDA-based RUL prediction.

### 2.1. Data Preprocessing

To achieve high accuracy in RUL prediction, standardisation of data should be regarded as the most important task, given the variances in capacity degradation data among different batteries. For battery capacity data to be standardized, two stages must be completed.

Step 1: Normalisation of data. The data on battery capacity are normalized to a range of 0–1, with the initial value being one and the failure threshold capacity being zero. This is because the battery capacity data tend to decrease gradually over time, from the initial value to the failure threshold, which in this study is set at 82% of the battery’s rated capacity.

Step 2: Normalisation of RUL labels. RUL labels represent the remaining life of capacity data and are also normalized to range from zero to one.

### 2.2. Similarity Measurement-Based Uncertainty Ellipse

**Figure 3:**
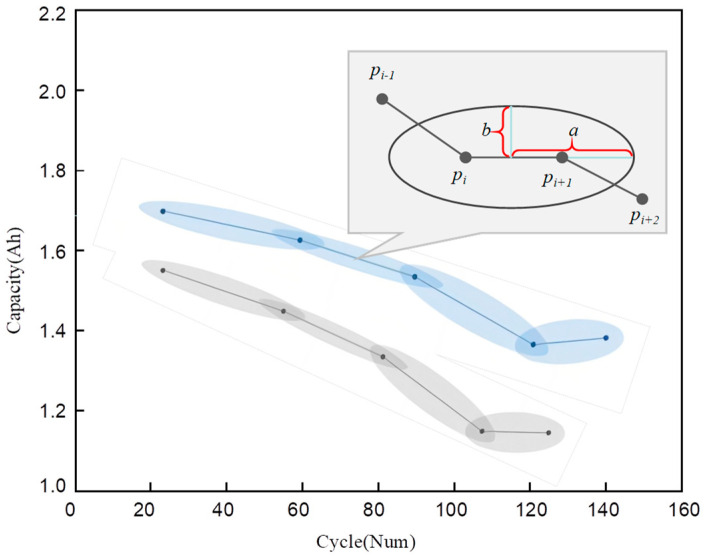
Schematic of degraded trajectories based on the ellipses model.

For uncertainty trajectory due to asynchronous sampling, the ellipse model has been proposed to characterize the likelihood of transferring paths between two sample points. As shown in Figure 3, the shape of the ellipse characterizes the uncertainty, and the size of the half-length axis b represents the magnitude of the uncertainty. In previous research, it has been conventionally assumed that the semi-major axis (b) is a deterministic quantity, determined by the maximum rate of the moving point [22]. However, it should be noted that, in practical scenarios, the degradation rate may vary among individuals due to inherent differences and environmental factors. Therefore, the value of b cannot be determined with certainty but is a variable with uncertainty. Unfortunately, our attempt to develop an accurate model for variable b was not feasible, as we faced difficulties in integrating these factors. Moreover, the lack of sufficient data impeded our capacity to produce an estimation using statistical analysis.

In order to address this issue, the most viable and efficient approach is to employ subjective evaluation methods that make optimal use of experts’ knowledge. The uncertain theory, introduced by Liu in 2007 [23], is posited as a novel mathematical tool that exhibits greater efficacy in addressing small-sample problems through expert knowledge, as opposed to probability theory. Therefore, we assume that the half-length axis *b is* an uncertain measure obeying a normal, uncertain distribution.

In uncertain theory, the uncertain measure satisfies the following axioms, assuming Γ is a nonempty set, ℒ is an algebra over, and ℳ is a set function:

Axiom 1 (Normality Axiom): ℳ{Γ}=1 for the universal set Γ.

Axiom 2 (Duality Axiom): $ℳ\{\Lambda \}+ℳ\{\Lambda^{c}\}=1$ for any event Λ∈ℒ.

Axiom 3 (Subadditivity Axiom): $ℳ\{\overset{\infty }{\underset{i=1}{\cup }}\Lambda_{i}\}\leq \sum_{i=1}^{\infty }ℳ\{\Lambda_{i}\}$

Axiom 4 (Product Axiom): $ℳ\{\prod_{k=1}^{\infty }\Lambda k\}=\Lambda_{k=1}^{\infty }{ℳ}_{k}\{\Lambda_{k}\}$

Returning to the focus of this article, we consider the half-length axis *b* as an uncertain variable that follows a normal distribution. It can be defined as:

(1) $$\Phi (x)={\left(1+\exp(\frac{\pi (e-x)}{\sqrt{3}\sigma })\right)}^{-1}$$

where Φ is the uncertainty distribution of *b*, and *x* could be any real number. The uncertainty distribution can be achieved through the utilization of expert knowledge. One possible approach to address this issue is to ask experts, “How likely is *b* less than or equal to *x* for the degradation trajectory of this product?” and utilize the expert’s answer as a belief degree (α). By iteratively posing the question with varying values of *x*, a collection of experimental data, denoted as $\left(x_{1},\alpha_{1}),(x_{2},\alpha_{2}),\ldots ,(x_{n},\alpha_{n}\right)$, can be obtained from experts. These data adhere to a consistency condition.

(2) $$\begin{array}{c} x_{1}<x_{2}<\ldots <x_{n-1}<x_{n}\\ 0<\alpha_{1}<\alpha_{2}<\ldots <\alpha_{n-1}<\alpha_{n}<1 \end{array}$$

After collecting the expert’s experimental data, we can employ the principle of least squares to estimate the parameters of the uncertainty distribution of *b*, which minimizes the sum of squares of the distance from the expert’s experimental data to the expected uncertainty distribution [24]. The objective function *Q* can be established as follows:

(3) $$Q=\sum_{i=1}^{K}(\Phi (x_{i})-\alpha_{i}^{*}{)}^{2}={\sum_{i=1}^{K}\left(\frac{1}{1+\exp(\frac{\pi (e-x_{i})}{\sqrt{3}\sigma })}-\alpha_{i}^{*}\right)}^{2}$$

**Figure 4:**
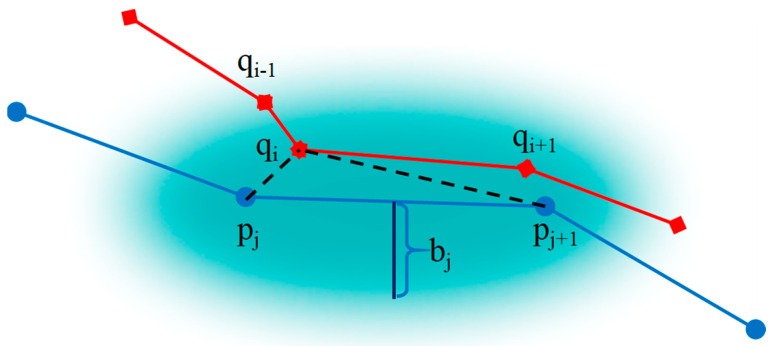
The similarity of uncertain trajectories.

In order to compute the similarity of uncertain trajectories based on the uncertain ellipse model, we define a new distance measure NS(P,Q). Just as Figure 4 shows, if a point $q_{i}$ in *Q* lies within the ellipse of the segment $\left(p_{j},p_{j+1}\right)$ in *P*, $q_{i}$ is matched to $\left(p_{j},p_{j+1}\right)$; otherwise, $q_{i}$ is mismatched to *P*.

Equation (4) calculates each point’s matching degree, where *q* is considered as NSP.

(4) $$s_{qi}=\exp\left(-\underset{f\in \{p_{j},p_{j+1}\}}{\min}\frac{d(q_{i},f_{o})}{a_{i}}\right)\cdot k(q_{i})$$

(5) $$k(q_{i})=\{\begin{array}{cc} \mathrm{current}\;\mathrm{length}\;\mathrm{of}\;\mathrm{continued}\;\mathrm{match} & q_{i}\;\mathrm{is}\;\mathrm{matched}\\ 0 & q_{i}\;\mathrm{is}\;\mathrm{mismatched} \end{array}$$

(6) $$a_{i}^{2}=b_{i}^{2}+c_{i}^{2}$$

(7) $$c=\frac{1}{2}d_{euc}(p_{j},p_{j+1})$$

where $S_{qi}$ denotes the matching degree of point $q_{i}$, and a and b are the main axes of the uncertain ellipse. $f_{0}$ is any focus of the ellipse. $k(q_{i})$ denotes the continuity coefficient, which reflects the present extent of the continued match. If the current point matches an ellipse from the other trajectory, its value will increase by one. It will be reset to zero when no ellipse matches the current point.

For each point in both trajectories, we compute its contribution to the overall similarity based on its position and matched uncertain ellipse. The unnormalized similarity can be calculated by summing the contribution of each point:

(8) $$S(P,Q)=\frac{1}{2}(\sum_{i=0}^{n-1}s_{qi}+\sum_{j=0}^{m-1}s_{pj})$$

Then, we can normalize the similarity measure results by dividing each contribution by the maximum continuity coefficient because the similarity contributions of a single point are multiplied by the continuity coefficient:

(9) $$NS(P,Q)=\frac{1}{2}(\sum_{i=0}^{n-1}s_{qi}/\sum_{i=1}^{n-1}i+\sum_{j=0}^{m-1}s_{ppj}/\sum_{j=1}^{m-1}j)$$

where *m* and *n* are the trajectories lengths of *P* and *Q*, respectively.

The algorithm for the uncertain matching model consists of two parts, as shown in Algorithm 1: sampling the shape parameters of the uncertain ellipse by a Monte Carlo method [25] and then computing the ULCS values by traversing a pair of trajectories. The overall computational complexity is O(*pq*), where *p* and *q* represent the lengths of different trajectories.
**Algorithm 1**: Uncertain measurement algorithm.Input: a pair of trajectories *P* and *Q*; Output: *NS*(*P*,*Q*): similarity value of *P* and *Q*;Step1: Generate a uniform random number b by uncertain distribution Φ(x)
Step2: Get the shape parameters of all *EL*(p) & *EL*(q) by Equations (6) and (7)Step3: For *q* in Q, traverse all the segments in trajectory P    Continutiy+ =1    If *q* is spatially located in the ellipse of segment (p), then    Sq+ = exp(−min(d(q,p_j_), d(q,p_j+1_))/EL(p) × Continuity    Repeat step 3 until fine the matched ellipse    Else    Continuity reset to 0 when the point is mismatchedStep4: Repeat the same operation from P to Q, get SpStep5: $S(P,Q)=1/2(\sum_{i=0}^{n-1}s_{qi}+\sum_{j=0}^{m-1}s_{pj})$    $NS(P,Q)=1/2(\sum_{i=0}^{n-1}s_{qi}/\sum_{i=1}^{n-1}i+\sum_{j=0}^{m-1}s_{pj}/\sum_{j=1}^{m-1}j)$


### 2.3. RUL Prediction Based on Stacked Denoising Autoencoder

**Figure 5:**
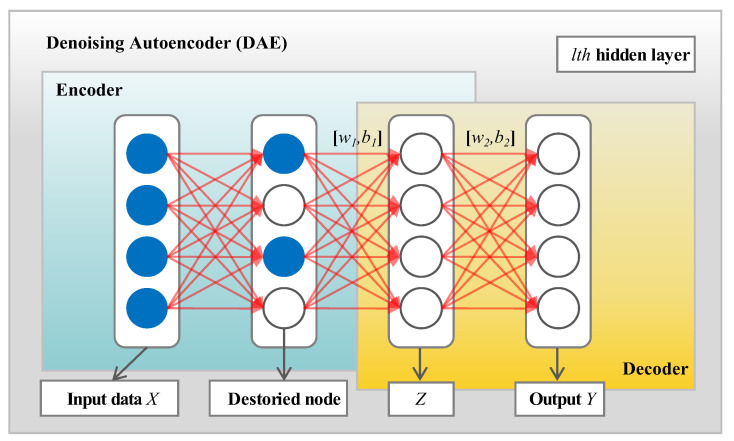
The basic structure of a DAE unit.

The SDA model is constructed by two Denoising Autoencoders (DAE). DAE is improved on the basis of the Autoencoder (AE) by adding noise to the original input of AE, which mainly includes two parts: the encoder and the decoder. The structure is depicted in Figure 5.

1. Encoder: The main role of the encoder is to convert inputs containing noise $X=\{x_{m}^{n}\}(1\leq m\leq S)$ to the nonlinear space $Z=\{z_{m}^{n}\}$ in the hidden layer by: $Z=f(W_{1}X+b_{1})$
  where $W_{1}$ is the connection weight matrix between the input layer and the hidden layer; $b_{1}$ is the bias matrix between the input layer and the hidden layer. *f* is the active sigmoid function: $\delta =1/(1+e^{-X})$.
2. Decoder: The decoder, on the other hand, is the inverse process of the Encoder, which converts the internal representation to a nonlinear output space *Y* using Equation (10). The output should be as close as possible to the original noise-free input:

(10) $$Y=f(W_{2}Z+b_{2})=f(W_{2}(W_{1}X+b_{1})+b_{2})=f(WX+b)$$

where $W_{2}$ and $b_{2}$ are the connection matrix and bias term between *Z* and *Y*, and ***W*** and ***b*** are the connection weight and bias term between the reconstructed output *Y* and input X. Similarly, *f* is the active sigmoid function.

DAE implements feature learning by minimizing the reconstruction error (loss function) *J* between the input data *X* and the output data *Y*, using a gradient descent algorithm to continuously adjust the network weights *W* and biases *b* to reduce the reconstruction error.

(11) $$J(W,b)=\frac{1}{N}\sum_{i=1}^{L}\frac{1}{2}{\left(Y-X\right)}^{2}$$

*L* represents the total number of hidden layers. (*W*,*b*) is repeatedly updated at the lth hidden layer using Equation (12):

(12) $$W_{l-1}=W_{l}-\eta \frac{\partial }{\partial W}J_{l-1}\;b_{l-1}=b_{l}-\eta \frac{\partial }{\partial b}J_{l-1}$$

where $\partial /\partial W(J_{l-1})$ represents the partial derivative of W in the *l*−1st hidden layer of the SGD algorithm and $\partial /\partial b(J_{l-1})$ is the partial derivative of b. η denotes the learning rate.

**Figure 6:**
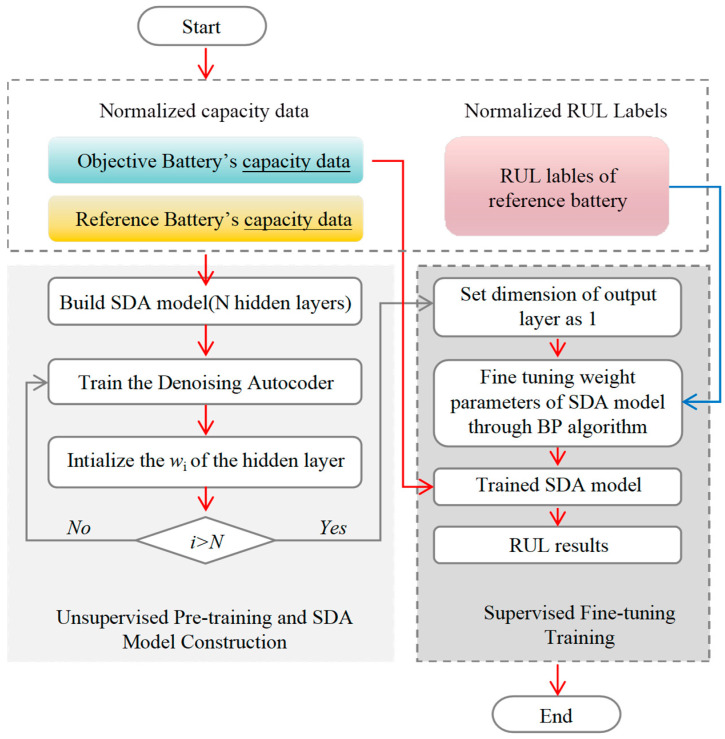
The training process of the SDA model.

The training process of SDA consists of two steps: (1) unsupervised pre-training and (2) supervised fine-tuning, as shown in Figure 6. The core idea of the pre-training process is to employ a level-by-level training scheme of SDA models to obtain a better representation of the input. Prior research has indicated that unsupervised pre-training yields superior training outcomes in comparison to the direct random selection of model parameters [26]. At this stage, only the capacity data of the reference battery is used as input data. Following the pre-training phase, the weight parameters of the SDA model are further improved by supervised learning, in which both the capacity of the reference battery and the RUL labels will be used as input data. The two-step training approach allows for the precise establishment of the complicated link between the normalized capacity data and the RUL labels of the reference battery. Finally, the trained SDA model is used to predict the RUL of the target battery by using the capacity of the objective battery.

## 3. Case Study

### 3.1. Battery Degradation Dataset

We assess the approach’s efficacy by employing both a synthetic and a real dataset about the degradation of lithium-ion batteries. The two datasets utilized in our experiments are characterized as follows:

(1) Real dataset: The dataset was provided by NASA Ames’s Prognostics CoE and contains a total of 18,650 degradation data for 34 groups of batteries. Although each type of battery has the same cathode and diaphragm materials, their anodes and electrolyte solutions are different. All of the above batteries were tested on a shared platform. The charging process was carried out using a constant current (CC) mode with a current of 1.5 A until the battery voltage reached the top limit of 4.2 V. Subsequently, the charging mode was switched to constant voltage (CV) until the charge current dropped to 20 mA. The battery underwent a controlled discharge process at a consistent current rate of 2 A until the voltage reached a level of 2.5 V. The dataset provides different data about battery aging, including the battery terminal voltage measured during the charge and discharge phases, battery output current measured during the charge and discharge phases, battery temperature, and battery capacity. Based on the preceding study of the existing performance measures for lithium-ion batteries, battery capacity was chosen as the degradation indicator. In this study, B0003, B0005, B0007, and B0010 (3#, 5#, 7#, and 10#) batteries are used to verify the proposed method. Number 10# is used as the objective battery; the others are used as reference batteries.

**Figure 7:**
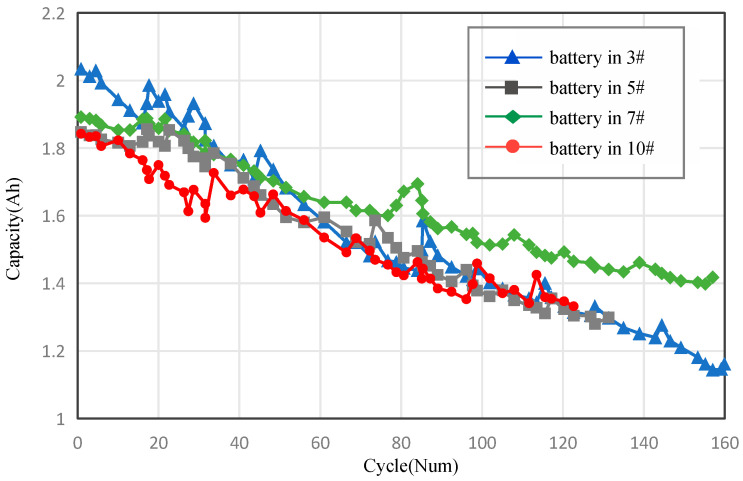
Degradation data for individual batteries in each group.

Figure 7 displays the degradation data for a single battery from each tested battery group. The battery capacity deteriorated considerably with an increase in the number of cycles. However, the degradation curves varied among various batteries.

**Figure 8:**
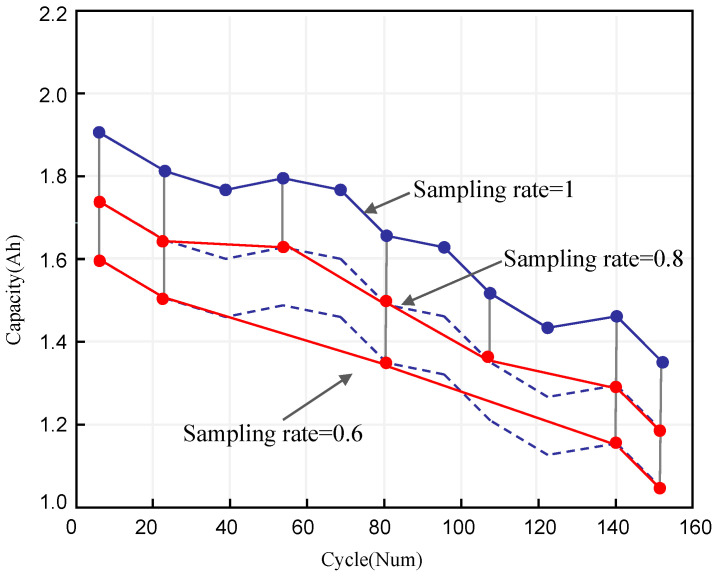
Synthetic datasets generated at different sampling rates. (Dashed line represents the original degradation trajectory).

(2) Synthetic dataset: The dataset was created by resampling real degradation data at various sampling rates. As depicted in Figure 8, sub-trajectories are generated for each trajectory ($T_{rr}$) by iteratively selecting points alternatingly. We define the new datasets as $D_{ra}^{\left(-\right)}=\{T_{raai}|i=1,2,\ldots ,n\}$, where *ra* represents the sampling rate, ranging from 1 to 0.6.

### 3.2. Similarity Measurement Result

Using the synthetic dataset $\left\{D_{ra=1}^{\left(-\right)},D_{ra=0.9}^{\left(-\right)},D_{ra=0.8}^{\left(-\right)},D_{ra=0.7}^{\left(-\right)},D_{ra=0.6}^{\left(-\right)}\right\}$, we compared our proposed method with traditional similarity metrics such as LCSS, DWT, and EDR.

(1) DTW [27]: Dynamic time warping (DTW) is a time series similarity matching algorithm based on dynamic programming. It offers significant benefits when matching time series of varying lengths.
(2) LCSS [28]: The Longest common subsequence (LCSS) defines the similarity between two trajectories as the longest common subsequence. LCSS is more robust to noise and permits specific points to remain unmatched, unlike DTW.
(3) EDR [29]: Edit Distance on Real Sequence (EDR) defines the similarity between two trajectories as the minimum number of operations to transform from one trajectory to another.

**Figure 9:**
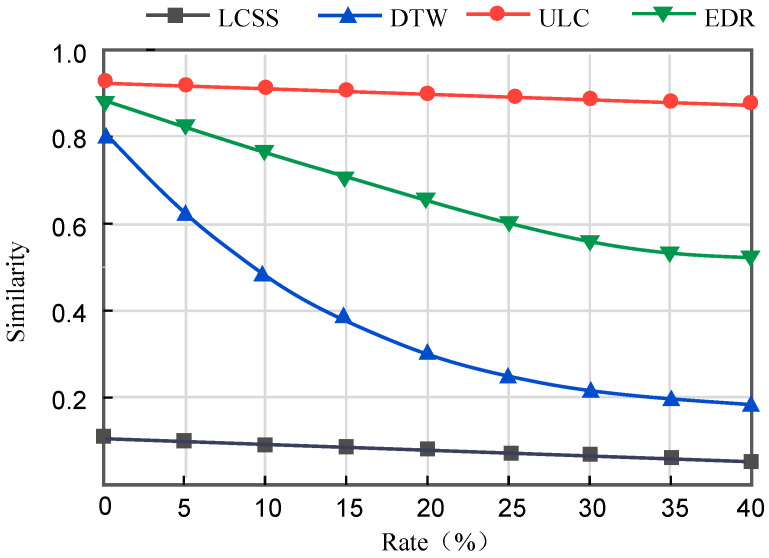
Similarity of trajectories resulting from decreasing sampling rate.

The outcomes of similarity across various sampling rates are illustrated in Figure 9. As the sampling rate decreases and the data of sampled points in the trajectory becomes sparser, the similarity calculated by all methods also decreases. Our proposed method continues to yield the most accurate similarity calculation results even at a sampling rate of 0.6, in contrast to the DTW method, which experiences a 60% decrease in accuracy, and the LCSS method, which experiences a 40% decrease. The LCSS yields a slight difference in results, mainly because it allows for partial point mismatches and is more robust to outliers. The cliff-like change in the DTW calculations is mainly due to a significant change in the shape of the trajectory as the sampling points become progressively sparser. The EDR method consistently achieves a low similarity computation result, potentially due to the algorithm’s high complexity, which mitigates the occurrence of errors. In summary, our proposed method exhibits superior performance compared to the other three methods. This observation serves as evidence for the efficacy of our approach in addressing the issue of uncertain trajectories.

### 3.3. Prediction Results and Comparison

**Figure 10:**
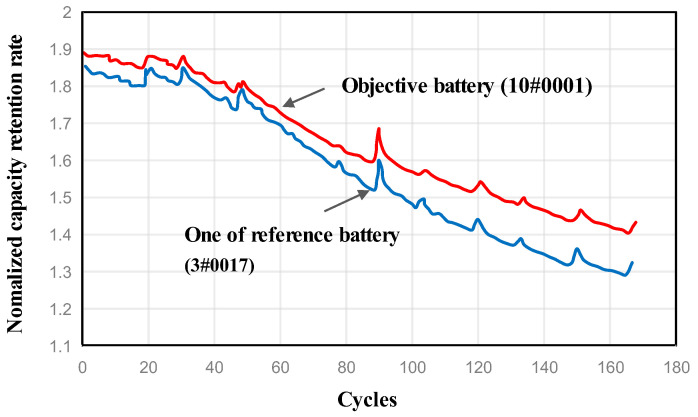
One of the most similar batteries was selected by the proposed method.

This part compares the RUL prediction results based on the actual dataset. We take battery 10# as the objective battery for analysis. The degradation curves of both the target battery and the reference battery selected by the proposed method are depicted in Figure 10. The figure demonstrates that the degradation curves exhibit a high degree of similarity in terms of degradation trend and distance, which, to some extent, proves the effectiveness of the new approach. After determining all the corresponding reference batteries through the calculation of similarity, the data is used to train the SDA-based prediction model.

**Figure 11:**
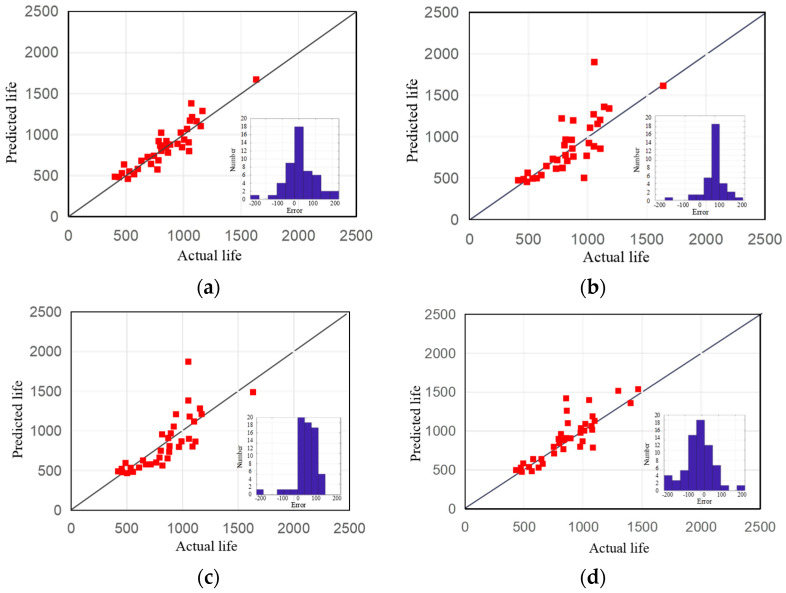
Predicted life and actual life and its error distribution: (**a**) *NS*(*P*,*Q*)-based; (**b**) DTW-based; (**c**) EDR-based; (**d**) LCSS-based.

Figure 11 depicts the prediction results, with each subgraph’s histogram illustrating the error distribution of actual and predicted battery lifetime.

For the performance metric, mean absolute percentage error (*MAPE*) and *R*^2^ are selected as the comparison criterion; the lower the value of *MAPE* and the higher the value of *R*^2^, the better the prediction result is.

(13) $$MAPE=\frac{100}{K}\sum_{k=1}^{K}\frac{\left|C_{k}-{\hat{C}}_{k}\right|}{C_{k}}$$

(14) $$R^{2}=1-\frac{\sum_{k=1}^{K}{\left(C_{k}-{\hat{C}}_{k}\right)}^{2}}{\sum_{k=1}^{K}{\left(C_{k}-{\bar{C}}_{k}\right)}^{2}}$$

where $C_{k}$, ${\hat{C}}_{k}$, and ${\bar{C}}_{k}$ represent the ground-truth capacity, estimated capacity, and the average of the truth capacity, respectively. *K* is the number of cycles.

Table 1 depicts the *MAPE* and *R*^2^ results of different models. Overall, the RUL prediction method incorporating epistemic uncertainty has produced superior results. Verification results indicate that the proposed method provides a workable solution to the epistemic uncertainty that pertains to the limitations of representing the motion between two sample points, which is influenced by the sampling rate. Regardless of data variations, the historical test database can be mined for the most comparable data, mitigating uncertainty’s effects. Additionally, combining with deep learning methods makes the prediction method excellent at modeling complex relationships with limited data and robust against data variations.

## 4. Conclusions

Reducing the uncertainty in Remaining Useful Life (RUL) predictions is of utmost importance due to its significant role in guiding various activities such as fault mitigation, fault recovery, and mission re-planning. In this paper, a new similarity-based RUL prediction methodology incorporating epistemic uncertainty is proposed. We propose a theory based on an uncertain ellipse model to resolve the epistemic uncertainty caused by sampling errors in trajectory data. By designating elliptical parameters as subject to an uncertain distribution, each trajectory segment corresponded to an uncertain ellipse that adaptively described the segment’s uncertainty region. Based on the model, the proposed similarity measure *NS*(*P*,*Q*) could depict similarity as a normalized value, effectively reflecting the impact of uncertainty. After selecting the degradation trajectories with the most significant similarity, the selected data are input into the SDA network to predict battery life cycles. Due to its layered structure and self-learning capability, the complex relationship between degradation data and RUL can be effectively learned. A comparison of the *MAPE* shows that the proposed method’s predictive performance was superior to that of DTW, EDR, and LCSS. Future efforts should concentrate on various fronts. This paper assumes that the parameters of the uncertainty ellipse follow a normal distribution of uncertainty, and the estimation of these parameters is conducted using empirical data-based uncertain theory. Given the diverse forms of parameter distribution types, future research should investigate the impact of various distribution types (e.g., triangular distribution) on similarity calculations.

## Figures and Tables

**Table 1:** Comparison of different models for RUL prediction.

Evaluate Criteria	*NS*(*P*,*Q*)-Based	LCSS-Based	DTW-Based	EDR-Based
*MAPE*	9.28	11.50	19.47	33.55
*R* ^2^	0.9226	0.7745	0.7298	0.4151

## Data Availability

The data presented in this study are available on request from the corresponding author.
